# Gene network expression of whole blood leukocytes in dairy cows with different milk yield at dry-off

**DOI:** 10.1371/journal.pone.0260745

**Published:** 2021-12-09

**Authors:** Luca Cattaneo, Matteo Mezzetti, Vincenzo Lopreiato, Fiorenzo Piccioli-Cappelli, Erminio Trevisi, Andrea Minuti

**Affiliations:** Department of Animal Sciences, Food and Nutrition (DIANA), Research Center Romeo and Enrica Invernizzi for sustainable dairy production (CREI), Facoltà di Scienze Agrarie, Alimentari e Ambientali, Università Cattolica del Sacro Cuore, Piacenza, Italy; INRA, FRANCE

## Abstract

Dairy cows at dry-off undergo several management and physiological changes, resulting in alterations in plasma biomarkers of inflammation, oxidative stress, and immune system. High milk yield at the end of lactation exacerbates these responses. The underlying mechanism of these changes has yet to be elucidated. We hypothesized altered leukocyte gene expression after dry-off and different responses in cows with different milk yield. Thirteen Holstein dairy cows were sampled at the turn of dry-off to investigated whole blood leukocyte gene expression and were grouped according to the average milk yield during the last week of lactation: low (< 15 kg/d) and high milk yield (> 15 kg/d). Blood samples were collected in PAXgene tubes (Preanalytix, Hombrechtikon, Switzerland) at -7, 7, and 34 days from dry-off (DFD) to measure mRNA abundance of 37 genes. Normalized gene abundance data were subjected to MIXED model ANOVA (SAS Institute Inc., Cary, NC). Compared with -7 DFD, at 7 DFD RNA abundance of lipoxygenase genes (*ALOX5*, *ALOX15*) and myeloperoxidase (*MPO*) increased, and that of the antioxidant gene (*SOD2*) decreased. Meanwhile, genes related to recognition and immune mediation (*CD16*, *MYD88*, *TLR2*), migration and cell adhesion (*CX3CR1*, *ITGAL*, *ITGB2*, *TLN1*), and the antimicrobial gene *MMP9* were downregulated at 7 or 34 DFD, whereas the antimicrobial *IDO1* gene was upregulated. Compared with low-producing cows, cows with high milk yield at dry-off cows had upregulated expression of the pro-inflammatory cytokines *IL8* and *IL18* and a greater reduction in transcript abundance of the toll-like receptor (TLR) recognition-related gene *TLR2*. Overall, the dry-off confirmed to be a phase of intense changes, triggering an inflammatory response and somewhat suppressing leukocyte immune function. In cows with high milk yield during the week before dry-off, the inflammatory response was exacerbated.

## Introduction

At dry-off, dairy cows have to face the transition from a lactating to a non-lactating state. After halting of milk removal, active mammary gland involution begins [1, 2] and many behavioral and physiological modifications happen [3, 4]. Milk synthesis stops, the mammary epithelium is partially renewed, different proteases are activated, and the permeability of tight junctions between epithelial cells increases [5]. Moreover, diet is changed and rumen papillae need to adapt [6]. Altogether, these changes affect metabolism, inflammation, and oxidative stress, even though at a lower degree than in the periparturient period [4]. In particular, blood NEFA increased immediately after dry-off, and concentrations of liver enzymes indicators, positive acute-phase proteins, and nitrogen species increased after dry-off, whereas negative APPs and antioxidant species decreased [4, 7]. Moreover, blood total leukocytes count decreased, mainly due to the reduction in neutrophils and monocytes [4, 7].

With the increase of genetic merit and the improvements in nutrition and management, cows that approach the scheduled dry-off day maintaining high milk yields are increasingly common [8]. In these cows, the metabolic and inflammatory response at dry-off is exacerbated and mammary gland involution is impaired [9]. Therefore, high milk production before dry-off represents a threat to cow’s udder health in the following lactation, and a safety threshold of 15 Kg/day has been proposed [9] In a previous study, Mezzetti et al. [7] observed that cows with an average milk yield above 15 Kg/day during the week before dry-off had an increased inflammatory response compared with those having a milk yield below this threshold.

Altered gene expression has been reported around calving in neutrophils [10] and leukocytes [11, 12], whereas the effects of dry-off on leukocytes gene expression have been poorly investigated. Nevertheless, insights on molecular changes of the immune cells at dry-off can provide information for a more accurate management of this fundamental physiological phase of the high-yielding dairy cow.

We hypothesized that leukocyte gene expression would differ around dry-off and between cows with high and low milk production before dry-off. Thus, we investigated the effect of dry off on genes involved in recognition, immune mediation, migration, cell adhesion, antimicrobial mechanisms, inflammatory cascade, oxidative stress, and the leukotriene pathway in leukocytes from cows with different milk yields during the week before dry-off.

## Materials and methods

### Animal management and PAXgene tubes sampling

All procedures were approved by the Università Cattolica Animal Welfare Committee and carried out in accordance with Italian laws on animal experimentation (DL n. 26, 04/03/2014) and ethics (Authorization of Italian Health Ministry N 1047/2015-PR). The trial was performed at the Università Cattolica del Sacro Cuore research dairy barn. The details about animal management and sampling procedure are described in previous work [7]. Briefly, 13 Holstein dairy cows (parity 1.9 ± 1.1; mean ± SD) were housed in individual tied stalls with controlled environmental conditions and milked twice daily until dry-off. Cows were abruptly dried off 55 days before the expected calving day and treated with an intramammary antibiotic and an injection of internal teat sealant (Mamyzin-A; Haupt Pharma Latina S.r.l, Italy). Before dry-off, cows were individually fed with the lactation diet. For 10 days after dry-off, cows were fed grass hay only. Afterward, dry period ration was administered. The diet composition was previously reported [7]. According to the average milk yield during the week before dry-off, cows were retrospectively divided into two groups, with a threshold of 15 Kg/day: low milk yield (**LM**; n = 7; 10.6 ± 3.7 Kg/d) and high milk yield (**HM**; n = 6; 16.5 ± 5.3 Kg/d). At -7, 7, and 34 days from dry-off (**DFD**), blood samples were collected through jugular venipuncture into PAXgene Blood RNA System tubes (Preanalytix, Hombrechtikon, Switzerland) for RNA extraction.

### RNA extraction, cDNA synthesis, and gene expression

RNA extraction from PAXgene tubes was performed according to the manufacturer’s protocol (Blood RNA Kit Handbook, PreAnalitix GmbH, Qiagen, Hilden, Germany), as described previously [11] and described in S1 Appendix. Afterward, RNA was quantified using the Qubit RNA BR Assay Kit (Invitrogen, Thermo Fisher Scientific, Waltham, MA), and RNA quality was assessed with the Experion Automated Electrophoresis System (Bio-Rad, Hercules, CA). The average RNA quality was 9.5 ± 0.6 (mean ± SD). Samples were diluted to 100 ng RNA/μL using nuclease-free water, and synthesis of cDNA was carried out with a reverse transcription kit (RevertAid RT Reverse Transcription Kit; Thermo Fisher Scientific). Diluted cDNA (4 μL) was combined with 6 μL of a 5 μL 1 × SYBR Green Master Mix (Applied Biosystems, Woolston Warrington, UK) + 0.4 μL each of 10 μM forward and reverse primers + 0.2 μL of nuclease-free water mixture., qPCR was performed with an Optical 384-Well Reaction Plate (CFX384 Touch; BioRad, Hercules, CA, USA), running three replicates for each sample. The qPCR efficiency and quantification cycle values were obtained for each reaction using LinReg-PCR (Version 2017.1; Amsterdam UMC, Amsterdam, the Netherlands). Genes selected for transcript analysis were those related to leukotrienes and oxidative status (*ALOX5*, *ALOX15*, *SOD1*, *SOD2*), inflammatory cascade (*CASP1*, *IL1B*, *IL1R*, *IL4*, *IL6*, *IL6R*, *IL10*, *IL18*, *IRAK1*, *IRAK4*, *NLRP3*, *S100A8*, *TNFRSF1A*, *TNF*), migration and cell adhesion (*CCR2*, *CD44*, *CX3CR1*, *IL8*, *ITGAL*, *ITGB2*, *LGALS8*, *SELL*, *SELPLG*, *TLN1*), recognition and immune mediation (*CD14*, *CD16*, *MYD88*, *TLR2*), and antimicrobial strategies (*IDO1*, *LCN2*, *MMP9*, *MPO*, *TLN2*). The final data were normalized using the geometric mean of three internal control genes: *ACTB*, *YWHAZ*, and *SDHA*. Gene names and functions, primer information, and primer sequencing results are included in Supporting tables. The stability of the normalization factor of these three control genes was assessed using GeNorm software and no improvement in stability was obtained with the addition of a fourth endogenous control gene.

### Statistical analysis

Normalized arbitrary mRNA abundance data were analyzed using the repeated measure mixed model, with the MIXED procedure of SAS version 9.4 (SAS Institute Inc., Cary, NC). The fixed effects were milk yield at dry-off (**MY**; LM and HM), sampling day (**DFD**; -7, 7, and 34), and their interaction (MY*DFD), whereas cows were included as random effect. All means were compared using the PDIFF statement of SAS and Dunnett’s adjustment was applied to compare sampling days (7 and 34 DFD) with the reference timepoint (-7 DFD). Significant differences were declared at *P* ≤ 0.05.

## Results and discussion

At dry-off, milking is stopped and mammary involution begin [4, 5], and, at the same time, energy content of the diet is dramatically reduced and rumen need to adapt [6, 13]. Therefore, the dry-off represents a potentially stressful event of the lactation cycle of dairy cows [14], with relevant effects on the subsequent lactation, which leads to huge alterations in plasma biomarkers of inflammation, metabolism, liver function, and oxidative stress [4, 7]. These responses are exacerbated in high-yielding dairy cows [7, 9]. Mammary gland gene expression is altered at the turn of dry-off [15, 16], but information about circulating leukocyte gene expression in this phase is lacking. Thus, we investigated the effect of dry off on peripheral blood leukocyte RNA abundance of genes involved in several pathways in cows with different average milk production during the last week before dry-off.

### Effects of dry-off on gene expression

In the present study, we evaluated mRNA abundance in circulating leukocytes. Blood leukocyte profile is affected by mammary involution, due to the migration of white blood cells to the mammary gland [4, 17, 18]. Therefore, some changes in mRNA abundance might be related to differential expression of genes in a specific leukocyte population. Dry-off affected (*P* < 0.05) transcript abundance of genes involved in inflammatory cascade (*NLRP3*), leukotriene regulation (*ALOX5*, *ALOX15*), recognition and immune mediation (*CD16*, *MYD88*, *TLR2*), migration and cell adhesion (*CX3CR1*, *ITGAL*, *ITGB2*, *TLN1*), antimicrobial strategies (*IDO1*, *MMP9*, *MPO*), and oxidative stress (*SOD2*).

**Fig 1:**
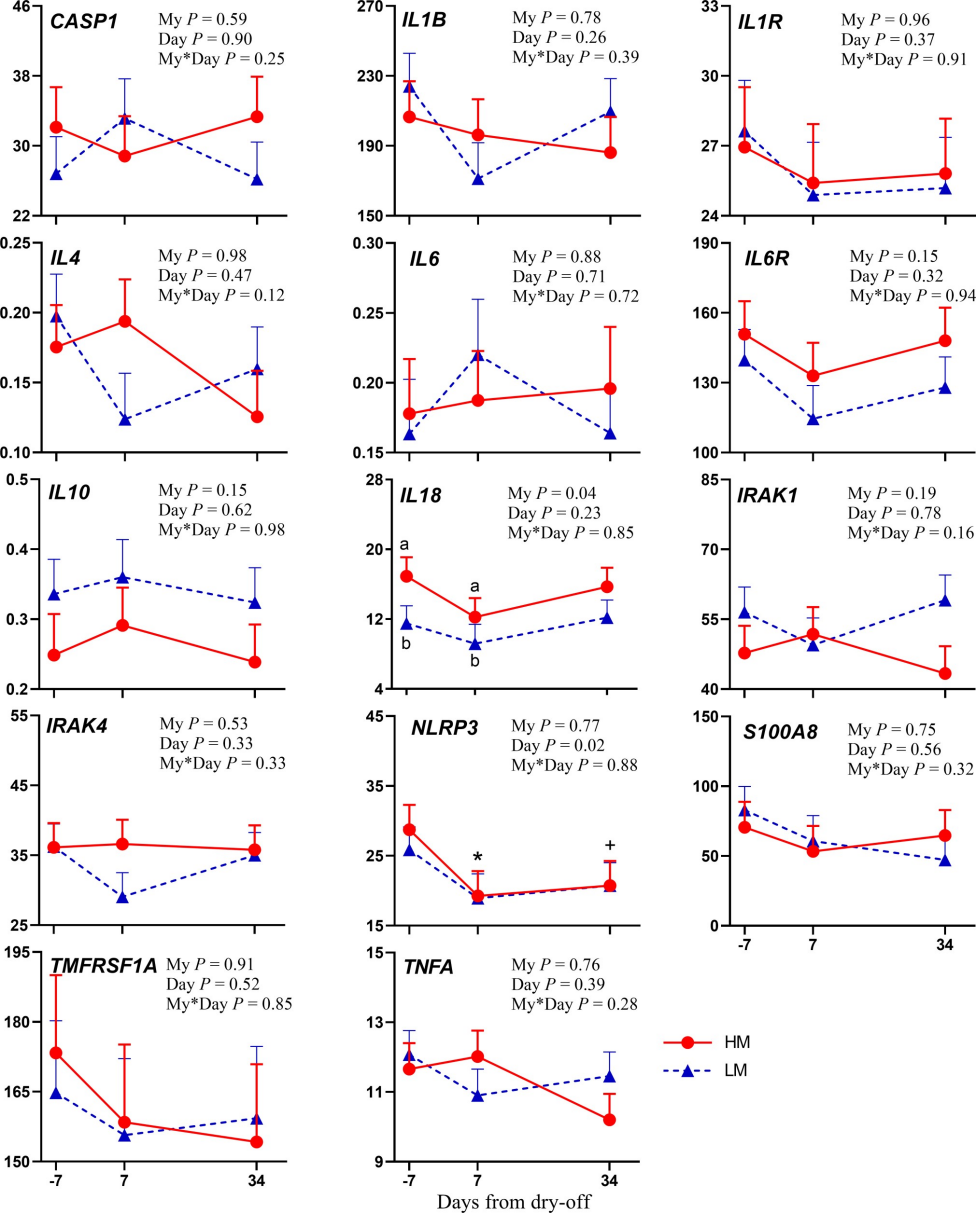
mRNA abundance of genes involved in the inflammatory cascade. Changes from -7 days from dry-off (DFD) to 34 DFD in dairy cows with high (HM; red line) or low (LM; blue line) milk yield at dry-off in mRNA abundance (mean ± SEM) for gene expression of genes involved in the inflammatory cascade: *CASP1* (Caspase 1), *IL1B* (Interleukin 1 Beta), *IL1R* (Interleukin 1 Receptor), *IL4* (Interleukin 4), *IL6* (Interleukin 6), *IL6R* (Interleukin 6 Receptor), *IL10* (Interleukin 10), *IL18* (Interleukin 18), *IRAK1* (Interleukin 1 Receptor-Associated Kinase 1), *IRAK4* (Interleukin 1 Receptor-Associated Kinase 4), *NLRP3* (NOD-Like Receptor Protein 3), *S100A8* (S100 Calcium Binding Protein A8), *TMFRSF1A* (TNF Receptor Superfamily Member 1A), *TNFA* (Tumor Necrosis Factor Alfa). *P*-values for main effect of milk yield at dry-off (My), day, and interaction of milk yield × day (My*Day) are shown. Significant differences (*P* ≤ 0.05) between groups on the same day are denoted with lowercase a and b, and differences between -7 DFD and 7 or 34 DFD are denoted with an asterisk (*; *P* ≤ 0.05) or a plus sign (+; *P* ≤ 0.1).

**Fig 2:**
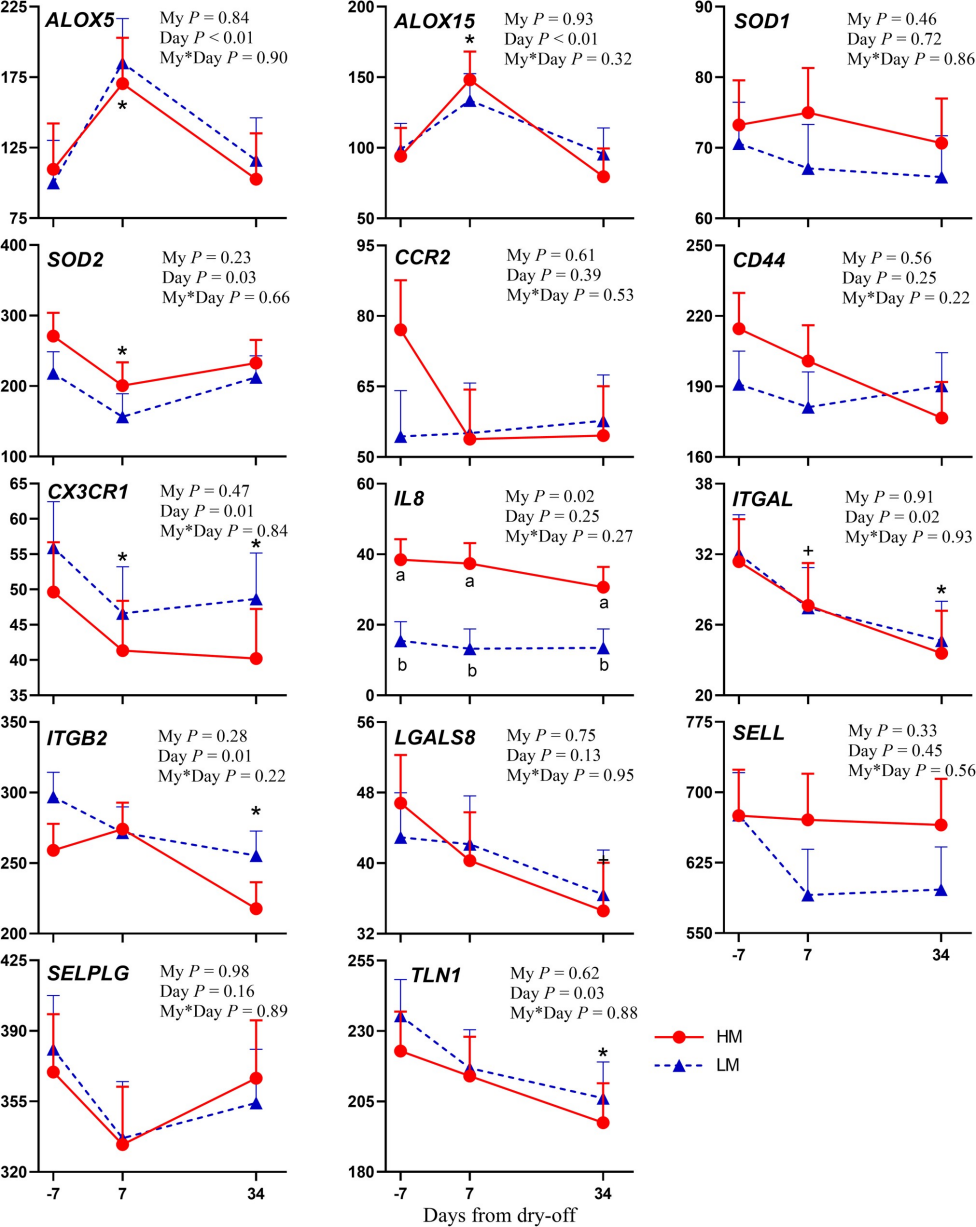
mRNA abundance of genes involved in leukotrienes and oxidative status pathways and migration and cell adhesion. Changes from -7 days from dry-off (DFD) to 34 DFD in dairy cows with high (HM; red line) or low (LM; blue line) milk yield at dry-off in mRNA abundance (mean ± SEM) for gene expression of genes involved in leukotrienes and oxidative status pathways and migration and cell adhesion: *ALOX5* (Arachidonate 5-Lipoxygenase), *ALOX15* (Arachidonate 15-Lipoxygenase), *SOD1* (Superoxide Dismutase 1), *SOD2* (Superoxide Dismutase 2), *CCR2* (C-C Chemokine Receptor Type 2), *CD44* (Hematopoietic Cell E- and L-Selectin Ligand), *CX3CR1* (CX3C Chemokine Receptor 1), *IL8* (Interleukin 8), *ITGAL* (Integrin Subunit Alpha L), *ITGB2* (Integrin Subunit Beta 2), *LGALS8* (Lectin, Galactoside-Binding, Soluble 8), *SELL* (Selectin L), *SELPLG* (Selectin P Ligand), *TLN1* (Talin 1). *P*-values for main effect of milk yield at dry-off (My), day, and interaction of milk yield × day (My*Day) are shown. Significant differences (*P* ≤ 0.05) between groups on the same day are denoted with lowercase a and b, and differences between -7 DFD and 7 or 34 DFD are denoted with an asterisk (*; *P* ≤ 0.05) or a plus sign (+; *P* ≤ 0.1).

The mRNA abundance of the gene encoding for NOD-like receptor protein 3 inflammasome (*NLRP3*) was reduced after dry-off (Fig 1). *NLRP3* is mainly expressed in monocytes and macrophages [19]. Its activation has been reported due to a variety of unrelated stimuli that induce cellular stress [20], and also by reactive oxygen species (ROS) [21], while nitric oxide inhibits the activation of the NLRP3 inflammasome [22]. We observed a reduced monocytes percentage on total leukocytes count (S6 Table) and an increased plasma concentration of nitric oxide were found after dry-off, probably accounting for the reduced *NLRP3* expression found in the present study. Arachidonate 5-Lipoxygenase (*ALOX5*) and arachidonate 15-Lipoxygenase (*ALOX15*) are genes involved in the leukotriene pathway and the inflammatory process. The enzyme *ALOX5* catalyzes the oxidation of arachidonic acid into leukotriene A4. It is increased during inflammation and is also involved in homeostasis restoration [23]. Through its pathway, it also produces hydroxyl and hydroperoxyl derivatives that are often elevated during inflammation [24]. *ALOX5* exists in the cytoplasm and nucleoplasm of cells, and its upregulation may occur during the maturation of leukocytes [25]. *ALOX15* plays a pivotal role in the resolution of inflammation [26], through the formation of key lipid mediators (e.g., lipoxins and resolvins) but through arachidonic acid metabolism also produces eicosanoids that act as pro-inflammatory mediators [27, 28] and are capable of generating ROS, metabolites strictly related to oxidative stress [29]. Therefore, their upregulation one week after dry-off (Fig 2) might suggest the activation of the inflammatory cascade after dry-off, but also the need for a modulatory mechanism that allows a rapid termination of the inflammatory process linked to the drastic changes taking place after milking cessation.

**Fig 3:**
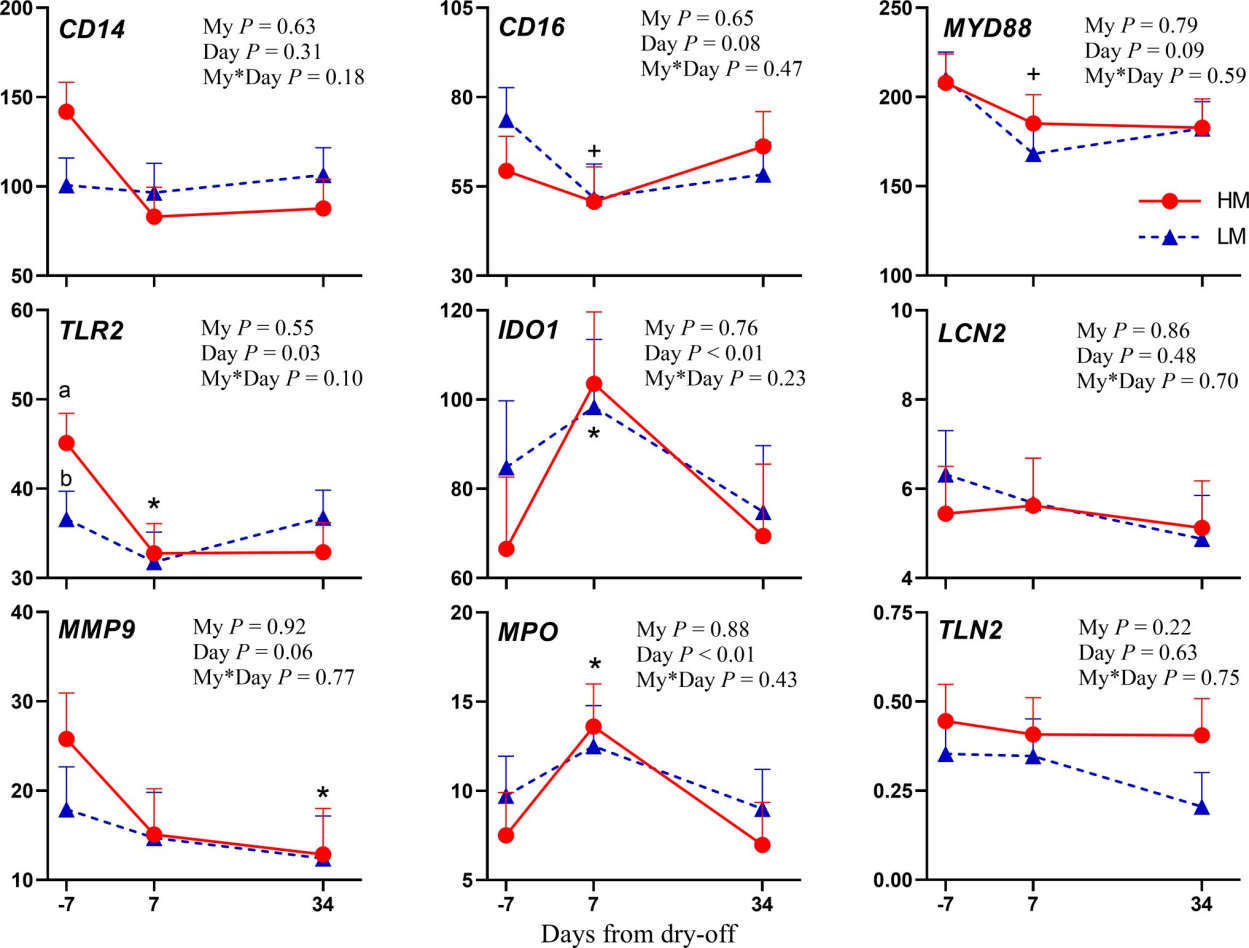
mRNA abundance of genes involved in recognition and immune mediation and antimicrobial strategies. Changes from -7 days from dry-off (DFD) to 34 DFD in dairy cows with high (HM; red line) or low (LM; blue line) milk yield at dry-off in mRNA abundance (mean ± SEM) for gene expression of genes involved in recognition and immune mediation and antimicrobial strategies: *CD14* (Cluster of Differentiation 14), *CD16* (Cluster of Differentiation 16 or Fc Fragment of Igg Receptor IIIa), *MYD88* (Myeloid Differentiation Primary Response Gene 88), *TLR2* (Toll-Like Receptor 2), *IDO1* (Indoleamine 2,3-Dioxygenase 1), *LCN2* (Lipocalin 2), *MMP9* (Matrix Metallopeptidase 9), *MPO* (Myeloperoxidase), *TLN2* (Talin 2). *P*-values for main effect of milk yield at dry-off (My), day, and interaction of milk yield × day (My*Day) are shown. Significant differences (*P* ≤ 0.05) between groups on the same day are denoted with lowercase a and b, and differences between -7 DFD and 7 or 34 DFD are denoted with an asterisk (*; *P* ≤ 0.05) or a plus sign (+; *P* ≤ 0.1).

The dry-off also affected the RNA abundance of Pathogen Associated Molecular Patterns -related genes (Fig 3). *CD16*, which is involved in the removal of the antigen-antibody complex from the circulation, had a lower abundance after dry-off, which can lead to a lower innate immune system efficiency against pathogens. However, CD16 is a cluster of differentiation molecule found on the surface of natural killer cells, neutrophils, monocytes, and macrophages [30]. The reduced *CD16* expression could be partially explained by the decrease in the number of these cells observed after dry-off [7] Similar responses were observed in genes involved in the toll-like receptors (TLRs) signaling, such as the myeloid differentiation primary response gene 88 (*MYD88*) and *TLR2*. TLRs recognize foreign non-self molecular products, initiating an inflammatory response against invading pathogens, consisting of alerting the body to infection, neutralizing pathogens, and repairing damaged tissues [31]. In particular, *TLR2* has bacterial peptidoglycan and lipoproteins as ligands [32]. After pathogens invasion, microbial products signal through TLRs on tissue-resident mast cells and macrophages, activate these cells to produce proinflammatory cytokines, which coordinates the recruitment of leukocytes together with the antimicrobial function [31]. *MYD88* acts as a signaling transductor of TLRs (not only of *TLR2*), by which is recruited [33]. The lower abundance during the week after dry-off of *TLR2* and *MYD88* might be a proxy of the suppression of the immune system activity in this phase. However, the exact reason why dry-off depressed immune system remains unknown. First, we analyzed RNA abundance in circulating leukocytes and their transcriptome could be different from that of milk or mammary tissue. We could hypothesize that the mammary gland has priority over other tissues during this phase of involution and remodeling, as is the case of the metabolic priority of the mammary gland in early lactation [34]. During involution, leukocyte concentration in the mammary gland increases [35], whereas decreased in the bloodstream [7], likely due to migration to the mammary gland. Moreover, blood phagocytic cells are more efficient than their milk counterparts [36, 37]. Therefore, even with a lower expression of related genes, they might be able to cope with systemic stimuli, at the same time prioritizing mammary gland immunity. Additionally, the switch from the high-energy diet of lactation to hay-feeding first and high-fiber dry period diet then might have played a role. Plasma NEFA concentration increased during the days following the dry-off [7] and they are known to have an immunosuppressive effect [38].

The suppression state of leukocyte immune function could be confirmed also by the genes related to migration and cell adhesion, that were downregulated after dry-off (Fig 2). Abrupt cessation of milking and the beginning of active involution leads to the recruitment of immune cells into the mammary gland [2, 18]. To avoid an excessive rate of migration, it would be possible to hypothesize that the migration capacity of circulating cells, not absorbed by the mammary gland, might be inhibited. A similar negative feedback mechanism was proposed for the control of neutrophils diapedesis and chemotaxis mediated by lysozyme in severe local inflammatory processes to prevent excessive tissue damage [39]. The protein CX3C chemokine receptor 1 (*CX3CR1*) is the receptor for CX3CL1, also known as fractalkine. It is expressed in immune and non-immune cells and their interaction mediates the chemotaxis of immune cells [40]. The combination of integrin alpha L chain (*ITGAL)* and the beta 2 chain (*ITGB2*) forms the lymphocyte function-associated antigen-1 (LFA-1), which has a relevant role in the extravasation of immune cells from the bloodstream to tissues [41]. In this case, the main target was probably the mammary gland, which faces huge challenges in the transition from milking to dry period. The cytoskeletal protein Talin-1 (*TLN1*) is also involved in neutrophil chemotaxis [42]. Together, the decrease in the mRNA abundance of these genes after dry-off might suggest a reduced leukocytes migration capacity, as observed in other studies after calving [10, 43]. Additionally, in the present study, the effect of these genes was maintained over one month after dry-off (34 DFD), likely indicating a persistency of this condition. Meanwhile, the antibiotic therapy at dry-off might have influenced these processes, even though, without a comparison with cows that did not receive the antibiotic therapy, we could not confirm this speculation. Alongside the immunosuppression noted after the dry-off, the antibiotic therapy may have reduced the bacterial load in the udder, causing the lack of immune system activation.

Moreover, dry-off, milk stasis, and mammary gland involution affected also genes involved in antimicrobial strategies (Fig 3). Indolamine 2,3-dioxygenase (*IDO1*) encodes a protein that catalyzes the degradation of the essential amino acid tryptophan, reducing its availability for pathogens at the site of infection [44]. Myeloperoxidase (*MPO*) release stimulates neutrophils killing of pathogens by phagocytosis or by antimicrobials release [45], catalyzes the production of hypochlorous acid [46], and induces neutrophils activation [47]. Interestingly, *MPO* was upregulated. The upregulation of both these genes shortly after dry-off might suggest a more active innate immune system in this phase, in order to cope with milk and pathogens stasis in the udder. These results are consistent with the increased lipoxygenase RNA abundance, suggesting the activation of inflammatory response after dry-off. Meanwhile, matrix metalloproteinase 9 (*MMP9*) abundance was lower after dry-off. *MMP9* is a collagenase of the gelatinase B group, which are zinc dependent proteinases degrading at least one component of the extracellular matrix or basement membrane [48]. In this way, they assist neutrophils’ migration from blood to the site of inflammation. Therefore, *MMP9* downregulation is consistent with that of genes involved in migration and cell adhesion (*CX3CR1*, *ITGAL*, *ITGB2*, and *TLN1*), and may be a signal of reduced immune cells migration capacity in the early dry period. The previously hypothesized negative feedback system, mediated by lysozyme or by another compound present in the mammary gland, might be implied. Opposite results have been reported in mammary dry secretions [49, 50], where *MMP9* dramatically increases during mammary involution, due to neutrophils infiltration and degranulation. Therefore, it seems that an increased immune system efficiency in the target site was paired with a reduced migration capacity into the bloodstream.

The dramatic changes at the turn of dry-off (i.e. diet change and abrupt milking cessation) resulted in oxidative stress, paired with an inflammatory response [7]. Superoxide dismutase (SOD) enzymes are one of the most efficient antioxidant systems, catalyzing the reduction of ROS [51]. In our study, *SOD2* abundance was reduced during the week after dry-off (Fig 2). A depletion of the antioxidant system was reported both after dry-off [7] and after calving [52]. Moreover, being *SOD2* the mitochondrial superoxide dismutase directly involved with the respiratory chain, its downregulation after dry-off might be related to the intense cell metabolism [53], typical of active mammary gland involution [2, 5]. Genes associated with oxidative stress, in particular in *SOD2*, were upregulated after dry-off [54]. However, in that research, the expression of alveolar tissue was analyzed, which is directly involved in mammary gland involution. In the present study, we analyzed blood leukocytes expression, and the different responses observed could be related to this important difference in the studies.

### Effects of milk yield at dry-off on gene expression

Milk yield before dry-off had a significant effect only on the abundance of genes involved in the inflammatory cascade. In fact, HM cows had increased peripheral leukocyte mRNA abundance of *IL18* and *IL8* related genes compared with LM (*P* = 0.04 and *P* = 0.02, respectively; Figs 1 and 2). *IL18* gene encodes a pro-inflammatory cytokine that enhances natural killer cell activity, the proliferation of activated T cells, and induces interferon-γ production from spleen cells, liver lymphocytes, and type-I T-helper cells [55], whereas *IL8* mediates the chemotaxis of neutrophils and other inflammatory cells from the blood into the mammary gland [56]. RNA abundance of *TLR2* decreased more markedly in HM cows (*P* = 0.10; Fig 3), likely suggesting a greater reduction in innate immune system activity in these cows. These results are consistent with previous findings of Mezzetti et al. [7], who observed increased inflammation after dry-off in higher producing cows, likely due to longer mammary tissue remodeling required.

## Conclusions

The dry-off alters blood biomarkers of nutrient metabolism, inflammation, and oxidative stress. This study investigated peripheral blood leukocyte RNA abundance of genes involved in pathways of inflammation, immune system, and oxidative stress. The dry-off triggered an inflammatory response and increased oxidative stress. Peripheral leukocytes antimicrobial and antioxidant capacity were somewhat impaired, but the exact reasons were unclear. In cows that produced more than 15 kg/day during the week before dry-off, the inflammatory response after dry-off was exacerbated. Therefore, the transition from lactation to the dry period needs special attention, in particular in high-yielding cows. Further research on gene expression would be needed in substrates closer to the mammary gland, such as the milk somatic cells, mammary epithelial cells, or mammary tissue.

## Supporting information

S1 Appendix RNA extraction protocol. Detailed description of RNA extraction from whole blood collected in PAXgene test tubes.
(DOCX)

S1 Table Genes of recognition and immune mediation. Target genes related to recognition and immune mediation functions and migration and cell adhesion with their biological function according to the National Center for Biotechnology Information (NCBI).
(DOCX)

S2 Table Genes of antimicrobial strategies, oxidative stress, and leukotrienes pathway. Target genes related to antimicrobial strategies, oxidative stress, and leukotrienes pathway with their biological function according to the National Center for Biotechnology Information (NCBI).
(DOCX)

S3 Table Genes of inflammatory cascade. Target genes related to the inflammatory cascade with their biological function according to the National Center for Biotechnology Information (NCBI).
(DOCX)

S4 Table PCR primers. GenBank accession number, sequence, and amplicon size of primers used to analyze gene expression by quantitative PCR.
(DOCX)

S5 Table Genes sequences. Sequencing results obtained from PCR product of Bos taurus specific primers for genes under investigation.
(DOCX)

S6 Table White blood cell percentages. Least square means of white blood cell populations percentage from -7 days from dry-off (DFD) to 34 DFD.
(DOCX)
