# Supplementary material for: Gene network expression of whole blood leukocytes in dairy cows with different milk yield at dry-off
Source: PLoS One. 2021 Dec 9;16(12):e0260745. doi: 10.1371/journal.pone.0260745 (PMC8659302; doi:10.1371/journal.pone.0260745)
Supplement: S1 Appendix — Detailed description of RNA extraction from whole blood collected in PAXgene test tubes. (DOCX) [file pone.0260745.s001.docx]

**S1 Appendix.** **RNA extraction protocol**

The complete procedure for RNA extraction from PAXgene RNA tubes were performed following the manufacturer’s protocol (Blood RNA Kit Handbook, PreAnalitix GmbH, Quiagen, Hilden, Germany). Nevertheless, some steps were modified according to previous trials carried out on bovine blood collected in PAXGene tubes. PAXgene tubes were incubated for a minimum of 2 h at room temperature (15–25 °C), in order to achieve the complete lysis of blood cells and then centrifuged for 10 min at 4000 x *g* using a swing-out rotor. The supernatant was removed by decanting and 4 mL of RNase-free water (PAXgene Blood RNA Kit, PreAnalitix GmbH, Quiagen, Hilden, Germany) was added to the pellet. Tubes were vortexed until the pellet was visibly dissolved and centrifuged for 10 min at 4000 x *g*. After discarding the supernatant, 350 μL of buffer BR1 (PAXgene Blood RNA Kit, PreAnalitix GmbH, Quiagen, Hilden, Germany) were added and then tubes were vortexed until the pellet was visibly dissolved. Samples were pipetted into 1.5 mL microcentrifuge tubes, 300 μL of buffer BR2 (PAXgene Blood RNA Kit, PreAnalitix GmbH, Quiagen, Hilden, Germany) and 40 μL proteinase K (PAXgene Blood RNA Kit, PreAnalitix GmbH, Quiagen, Hilden, Germany) were added. Samples were vortexed and incubated for 10 minutes at 55 °C using a shaker–incubator at 1200 rpm. The lysate was pipetted into a PAXgene Shredder spin column (PAXgene Blood RNA Kit, PreAnalitix GmbH, Quiagen, Hilden, Germany) and placed in a 2 mL processing tube. Tubes were centrifuged 3 min at 20,000 x *g*. The entire supernatant of the flow-through fraction was transferred to a new 1.5 mL microcentrifuge tube without disturbing the pellet in the processing tube and added 350 μL of ethanol (96–100 %; Sigma-Aldrich, Darmstadt, Germany). Samples were vortexed and centrifuged for 2 sec at 1000 x *g* in order to remove drops from the inside of the tube lid. 700 μL of sample were transferred into the PAXgene RNA spin column (PAXgene Blood RNA Kit, PreAnalitix GmbH, Quiagen, Hilden, Germany) placed in a 2 mL processing tube, and centrifuged for 1 min at 20,000 x *g*. The spin column was placed in a new 2 mL processing tube, and the old one containing flow-through was discarded. 350 μL of Buffer BR3 (PAXgene Blood RNA Kit, PreAnalitix GmbH, Quiagen, Hilden, Germany) were added into the PAXgene RNA spin column, centrifuged for 1 min at 20,000 x *g*, and placed in a new 2 mL processing tube, discarding the old processing tube containing flow-through. DNase I incubation mix (80 μL; prepared according to the kit handbook instructions; PAXgene Blood RNA Kit, PreAnalitix GmbH, Quiagen, Hilden, Germany) was pipetted directly onto the PAXgene RNA spin column membrane, and tubes were placed on the benchtop (20–30 °C) for 15 min. 350 μL of Buffer BR3 were added into the PAXgene RNA spin column and centrifuged for 1 min at 20,000 x *g*. After that, the spin column was placed in a new 2 mL processing tube, discarding the old processing tube containing flow- through. 500 μL of Buffer BR4 (PAXgene Blood RNA Kit, PreAnalitix GmbH, Quiagen, Hilden, Germany) were added into the PAXgene RNA spin column and centrifuged for 1 min at 20,000 x *g*. Spin column was then placed in a new 2 mL processing tube, discarding the old processing tube containing flow-through. Other 500 μL of Buffer BR4 were added to the PAXgene RNA spin column, following a centrifugation for 3 min at 20,000 x *g*. Tube containing the flow-through was discarded and PAXgene RNA spin column was placed in a new 2 mL processing tube. Tubes were centrifuge for 1 minute at 18,000 x *g* with lid opened. After discarding the processing tube containing the flow-through, PAXgene RNA spin column was placed in a 1.5 mL microcentrifuge tube, and 35 μL of Buffer BR5 (elution buffer; PAXgene Blood RNA Kit, PreAnalitix GmbH, Quiagen, Hilden, Germany) were pipetted directly onto the PAXgene RNA spin column membrane. Tubes were centrifuge for 1 min 12,000 x *g* in order to elute the RNA.
