# Supplementary material for: Gene network expression of whole blood leukocytes in dairy cows with different milk yield at dry-off
Source: PLoS One. 2021 Dec 9;16(12):e0260745. doi: 10.1371/journal.pone.0260745 (PMC8659302; doi:10.1371/journal.pone.0260745)
Supplement: S1 Table — Target genes related to recognition and immune mediation functions and migration and cell adhesion with their biological function according to the National Center for Biotechnology Information (NCBI). (DOCX) [file pone.0260745.s002.docx]

| **S1 Table. Genes of recognition and immune mediation****.** Target genes related to recognition and immune mediation functions and migration and cell adhesion with their biological function according to the National Center for Biotechnology Information (NCBI). | |
| --- | --- |
| **Gene symbol** | **Gene name and function** |
| *CCR2* | C-C chemokine receptor type 2 |
|  | Chemokine which specifically mediates monocyte chemotaxis. |
| *ITGB2* | Integrin Subunit Beta 2 |
|  | Integrins are cell-surface proteins that participate in cell adhesion as well as cell-surface mediated signaling. |
| *TLN1* | Talin 1 |
|  | It distributes with integrins in the cell surface membrane in order to assist in the attachment of adherent cells to extracellular matrices and of lymphocytes to other cells. |
| *TLN2* | Talin 2  It is a protein related to talin 1, a cytoskeletal protein that plays a significant role in the assembly of actin filaments and in spreading and migration of various cell types, including fibroblasts and osteoclasts |
| *ITGAL* | Integrin Subunit Alpha L |
|  | Combines with the beta 2 chain (ITGB2) to form the integrin lymphocyte function-associated antigen-1 (LFA-1) expressed in all leukocytes and a central role in leukocytes intercellular adhesion. |
| *CX3CR1* | C-X3-C Motif Chemokine Receptor 1 |
|  | Encoding for a fractalkine-receptor, transmembrane protein and chemokine involved in the adhesion and migration of leukocytes. |
| *SELL* | Selectin L |
|  | Encodes a cell surface adhesion molecule belonging to a family of adhesion/homing receptors. |
| *SELPLG* | Selectin P Ligand |
|  | Encodes a glycoprotein that functions as a high affinity counter-receptor for the cell adhesion molecules P-, E- and L-selectin expressed on myeloid cells and stimulated T lymphocytes. |
| *CD14* | Cluster of Differentiation 14 |
|  | Surface antigen preferentially expressed on monocytes/macrophages that binds bacterial-LPS in concert with LBP and mediating the innate immune response. |
| *CD16* | Cluster of differentiation 16 or Fc Fragment of IgG Receptor IIIa |
|  | Expressed on NK cells, this gene encodes for the Fc portion of IgG involving in the removal of antigen-antibody complexes from the circulation. |
| *CD44* | Hematopoietic Cell E- And L-Selectin Ligand |
|  | The protein encoded by this gene is a cell-surface glycoprotein involved in cell-cell interactions, cell adhesion and migration. |
| *LGALS8* | Lectin, Galactoside-Binding, Soluble 8 (Galectin 8) |
|  | Member of the galectin family. Involved in development, differentiation, cell-cell adhesion, cell-matrix interaction, growth regulation, apoptosis, and RNA splicing. |
| *MYD88* | Myeloid Differentiation Primary Response 88 |
|  | It encodes for a cytosolic adapter protein with a central role in the innate and adaptive immune response. Essential signal transducer in the IL-1 and TLR signaling pathways leading to the activation of numerous pro-inflammatory genes. |
| *TLR2* | Toll Like Receptor 2 |
|  | Encoding for a protein member of TLR family playing a fundamental role in pathogen recognition and activation of innate immunity. Activation of TLRs by PAMPs (pathogen-associated-molecular-patterns) results in an up-regulation of pathways to modulate the inflammatory response. |
|  |  |
