# Supplementary material for: Gene network expression of whole blood leukocytes in dairy cows with different milk yield at dry-off
Source: PLoS One. 2021 Dec 9;16(12):e0260745. doi: 10.1371/journal.pone.0260745 (PMC8659302; doi:10.1371/journal.pone.0260745)
Supplement: S2 Table — Target genes related to antimicrobial strategies, oxidative stress, and leukotrienes pathway with their biological function according to the National Center for Biotechnology Information (NCBI). (DOCX) [file pone.0260745.s003.docx]

| **S2 Table. Genes of antimicrobial strategies, oxidative stress, and leukotrienes pathway.** Target genes related to antimicrobial strategies, oxidative stress, and leukotrienes pathway with their biological function according to the National Center for Biotechnology Information (NCBI). | |
| --- | --- |
| **Gene symbol** | **Gene name and function** |
| *MMP9* | Matrix Metallopeptidase 9 |
|  | Involved in IL-8-indiced mobilization of hematopoietic progenitor cells from bone marrow but also involved in the breakdown of extracellular matrix (IV and V collagens) upon activation by extracellular proteinases. |
| *MPO* | Myeloperoxidase |
|  | This gene encodes for the enzyme myeloperoxidase, a heme protein synthesized during myeloid differentiation and constituting the major component of neutrophil azurophilic granules. This enzyme produces hypothallus acids central to the microbicidal activity of neutrophils. |
| *LCN2* | Lipocalin 2 |
|  | The gene encodes for a protein neutrophil gelatinase-associated lipocalin and plays a role in innate immunity by limiting bacterial growth via sequestering iron-containing siderophores. |
| *IDO1* | Indoleamine 2,3-Dioxygenase 1 |
|  | The enzyme encoded by this gene catalyzes the first and rate limiting step of the catabolism of the essential amino acid tryptophan along the kynurenine pathway. Limits the growth of intracellular pathogens by depriving tryptophan. In addition, tryptophan shortage inhibits T lymphocytes division and accumulation of tryptophan catabolites induces T-cell apoptosis and differentiation of regulatory T-cells. |
| *SOD1* | Superoxide Dismutase 1 |
|  | The protein encoded by this gene binds copper and zinc ions and is one of two isozymes responsible for destroying free superoxide radicals in the body. The encoded isozyme is a soluble cytoplasmic protein, acting as a homodimer to convert naturally-occurring but harmful superoxide radicals to molecular oxygen and hydrogen peroxide. |
| *SOD2* | Superoxide Dismutase 2 |
|  | This gene is a member of the iron/manganese superoxide dismutase family. It encodes a mitochondrial protein that forms a homotetramer and binds one manganese ion per subunit. This protein binds to the superoxide byproducts of oxidative phosphorylation and converts them to hydrogen peroxide and diatomic oxygen. |
| *ALOX5* | Arachidonate 5-Lipoxygenase |
|  | The encoded protein catalyzes the conversion of arachidonic acid to 5(S)-hydroperoxy-6-trans-8,11,14-cis-eicosatetraenoic acid, and further to the allylic epoxide 5(S)-trans-7,9-trans-11,14-cis-eicosatetrenoic acid (leukotriene A4). |
| *ALOX15* | Arachidonate 15-Lipoxygenase |
|  | The encoded enzyme acts on various polyunsaturated fatty acid substrates to generate various bioactive lipid mediators such as eicosanoids, hepoxilins, lipoxins, and other molecules. The encoded enzyme and its reaction products have been shown to regulate inflammation and immunity. |
|  |  |
