# Supplementary material for: Gene network expression of whole blood leukocytes in dairy cows with different milk yield at dry-off
Source: PLoS One. 2021 Dec 9;16(12):e0260745. doi: 10.1371/journal.pone.0260745 (PMC8659302; doi:10.1371/journal.pone.0260745)
Supplement: S3 Table — Target genes related to the inflammatory cascade with their biological function according to the National Center for Biotechnology Information (NCBI). (DOCX) [file pone.0260745.s004.docx]

| **S3 Table. Genes of inflammatory cascade.** Target genes related to the inflammatory cascade with their biological function according to the National Center for Biotechnology Information (NCBI). | |
| --- | --- |
| **Gene symbol** | **Gene name and function** |
| *IL1R1* | Interleukin 1 Receptor Type 1 |
|  | It encodes a cytokine receptor for IL-1 alpha, beta, and IL-1 receptor antagonist. It represents an important mediator involved in many cytokine-induced immune and inflammatory responses. |
| *IL4* | Interleukin 4  Pleiotropic cytokine produced by activated T cells. It is a ligand for interleukin 4 receptor that also binds to IL13, which may contribute to many overlapping functions of this cytokine and IL13. STAT6, a signal transducer and activator of transcription, has been shown to play a central role in mediating the immune regulatory signal of this cytokine |
| *IL6* | Interleukin 6  Cytokine that functions in inflammation and the maturation of B cells and as been shown to be an endogenous pyrogen capable of inducing fever in people with autoimmune diseases or infections. |
| *IL6R* | Interleukin 6 Receptor  IL6 receptor is a protein complex consisting of this protein and interleukin 6 signal transducer (IL6ST/GP130/IL6-beta), a receptor subunit also shared by many other cytokines |
| *IL8* | Interleukin 8 |
|  | Chemokine protein with major functions as mediator of inflammatory response and chemoattractant for immune cells. |
| *IL10* | Interleukin 10  Cytokine produced primarily by monocytes and to a lesser extent by lymphocytes. It down-regulates the expression of Th1 cytokines, MHC class II Ags, and costimulatory molecules on macrophages. |
| *IL18* | Interleukin 18 |
|  | The cytokine encoded is a pro-inflammatory cytokine that enhances NK cell activity in spleen cells and stimulates IF-gamma production in T-helper type I cells. |
| *IRAK1* | Interleukin 1 Receptor Associated Kinase 1 |
|  | The protein encoded represents one of two putative serine/threonine kinases that become associated with IL1R upon stimulation. Partially responsible for IL1-induced upregulation of the transcription factor NF-kappa B. |
| *IRAK4* | Interleukin 1 Receptor Associated Kinase 4  Serine/threonine-protein kinase that plays a critical role in initiating innate immune response against foreign pathogens. Involved in Toll-like receptor (TLR) and IL-1R signaling pathways. |
| *NLRP3* | Nucleotide-Binding Oligomerization Domain, Leucine Rich Repeat |
|  | Member of the NALP3 inflammasome complex. This complex acts as an upstream activator of NF-kappa B signaling and it plays a role in the regulation of inflammation, immune response, and apoptosis. |
| *S100A8* | S100 Calcium Binding Protein A8 |
|  | Member of the S100 family of proteins containing 2 EF-hand calcium-binding motifs. Involved in the regulation of cell cycle progression and differentiation. Prominent role also in the regulation of inflammatory processes and immune response. It can induce neutrophil chemotaxis and adhesion. |
| *CASP1* | Caspase 1 |
|  | Protein encoded from this gene activates the inactive precursor of IL-1. Furthermore, induce cell apoptosis. |
| *TNF* | Tumor Necrosis Factor Alpha |
|  | Pro-inflammatory cytokine belonging to TNF superfamily. Secreted mainly by macrophages, this cytokine is involved in the cell proliferation, differentiation, apoptosis, lipid metabolism, and coagulation. |
| *TNFRSF1A* | TNF Receptor Superfamily Member 1A  Membrane-bound and soluble forms of receptor encoded by this gene interact with  TNF-alpha. Binding of membrane-bound TNF-alpha to the membrane-bound receptor  induces activation, playing a role in cell survival, apoptosis, and inflammation. |
| *IL1B* | Interleukin 1 Beta |
|  | The cytokine encoded by this gene is produced by activated macrophages and it is proteolytically activated by CASP1. Key mediator of the inflammatory response and involved in cell proliferation, differentiation, and apoptosis. |
