# Supplementary material for: Gene network expression of whole blood leukocytes in dairy cows with different milk yield at dry-off
Source: PLoS One. 2021 Dec 9;16(12):e0260745. doi: 10.1371/journal.pone.0260745 (PMC8659302; doi:10.1371/journal.pone.0260745)
Supplement: S4 Table — GenBank accession number, sequence, and amplicon size of primers used to analyze gene expression by quantitative PCR. (DOCX) [file pone.0260745.s005.docx]

| **S4 Table. PCR primers.** GenBank accession number, sequence and amplicon size of primers used to analyze gene expression by quantitative PCR. | | | | |
| --- | --- | --- | --- | --- |
| **Accession no.** | **Gene** | **Primers^1^** | **Primers (5’-3’)** | **(bp)^2^** |
| XM_015459671.1 | *CCR2* | F.138 | CGTGGGACAAATCGAAGCAC | 199 |
|  |  | R.336 | AGCATAGTGAGCCCAGAACG |  |
| NM_174013.3 | *CD44* | F.752 | GAAGCACTTCAGGAGGCTACA | 125 |
|  |  | R.876 | GCCGTAGTCTCTGGTATCCG |  |
| NM_001102558.2 | *CX3CR1* | F.75 | CCCAGCCCAGGTGCTCA | 175 |
|  |  | R.249 | CAGCAAATTTCCCACCAGGC |  |
| NM_198221.2 | *ITGAL* | F.1582 | ATCAACGGGGATGAGCTGAC | 126 |
|  |  | R.1707 | GGTCCCTTCTATCCGCTGAC |  |
| NM_175781.1 | *ITGB2* | F.1231 | GACACCCTGAAAGTCACCTACGA | 108 |
|  |  | R.1338 | GAAGGTGATCGGGACGTTGAT |  |
| NM_174744.2 | *MMP9* | F.1169 | GCCCGGATCAAGGATACAGC | 128 |
|  |  | R.1296 | GGGGTGCTCCTCTGTGAATC |  |
| NM_174182.1 | *SELL* | F.588 | CTCTGCTACACAGCTTCTTGTAAACC | 104 |
|  |  | R.691 | CCGTAGTACCCCAAATCACAGTT |  |
| NM_001037628.2 | *SELPLG* | F.123 | CTGAGCACGGTGCCATGTTTC | 111 |
|  |  | R.233 | CTGGGGCCTTCACAGTTTCA |  |
| BC146107.1 | *TLN1* | F.942 | TTCCTGCCCAAGGAGTATGTG | 100 |
|  |  | R.1041 | AGCGTACCTTGGCCTCAATCT |  |
| XM_024997998.1 | *TLN2* | F.978 | GGCTGGATCACAGTCGAACA | 144 |
|  |  | R.1071 | CCCGAGCCTGAACATAGAGC |  |
| NM_174008.1 | *CD14* | F.525 | TCCGTAACGTATCGTGGACAAC | 100 |
|  |  | R.624 | GAGTGTGCTTGGGCAATGTTC |  |
| NM_001077402.1 | *CD16* | F.252 | CAGGACAGTGGCGAGTACAAGT | 100 |
|  |  | R.351 | GAGCGACCTGGAGCAATAGC |  |
| XM_015460928.1 | *LGALS8* | F.576 | CCCGGTCATCCCCTATGTTG | 100 |
|  |  | R.675 | CTGGAACCTGTCCGAGTCAC |  |
| NM_001014382.2 | *MYD88* | F.367 | GGAGGACTGCCAAAAGTATATTCTG | 105 |
|  |  | R.471 | GCCATGTCATTTATCCGAGTTATG |  |
| XM_003586675.4 | *LCN2* | F.749 | CCAGTGAGCCTGCACCTTTG | 141 |
|  |  | R.889 | TATTTAGCAGGCAAGGCAGGG |  |
| NM_001113298.2 | *MPO* | F.1311 | AGCCATGGTCCAGATCATCAC | 105 |
|  |  | R.1415 | ACCGAGTCGTTGTAGGAGCAGTA |  |
| NM_174615.2 | *SOD1* | F.256 | GGCTGTACCAGTGCAGGTCC | 101 |
|  |  | R.356 | GCTGTCACATTGCCCAGGT |  |
| NM_201527.2 | *SOD2* | F.620 | TGTGGGAGCATGCTTATTACCTT | 95 |
|  |  | R.714 | TGCAGTTACATTCTCCCAGTTGA |  |
| NM_001113725.2 | *S100A8* | F.19 | ATTTTGGGGAGACCTGGTGG | 124 |
|  |  | R.142 | ACGGCGTGGTAATTCCCTTT |  |
| NM_001101866.2 | *IDO1* | F.18 | ACTGCAAGAATGGCAGGTGA | 125 |
|  |  | R.142 | GGATGAGGTAGGTCCTCCAGT |  |
| NM_174197.2 | *TLR2* | F.3182 | CCATGTCTGGAGAGGGTGTT | 102 |
|  |  | R.3283 | GGGGACACAAAACAGCACTT |  |
| NM_174814.2 | *YWHAZ* | F. | TGAAAATGAAAGGAGACTACTACCG | 84 |
|  |  | R. | GCTGTGACTGGTCCACAATC |  |
| NM_173979.3 | *ACTB* | F.258 | ACCAACTGGGACGACATGGA | 149 |
|  |  | R.406 | GTCTCGAACATGATCTGGGTCAT |  |
| NM_174178.2 | *SDHA* | F. | CTGAAGCAGGTTTCAACACG | 113 |
|  |  | R. | GTTGTCCTCCTCCATGTTCC |  |

^1^ Primer direction (F – forward; R – reverse) and hybridization position on the sequence. are underlined.

^2^ Amplicon size in base pair (bp).

| **S4 Table (cont.).** GenBank accession number, sequence and amplicon size of primers used to analyze gene expression by quantitative PCR. | | | | |
| --- | --- | --- | --- | --- |
| **Accession no.** | **Gene** | **Primers^1^** | **Primers (5’-3’)** | **(bp)^2^** |
| NM_001192792 | *ALOX5* | F.771 | GCAGGAAGACCGCATGTTTG | 163 |
|  |  | R.933 | GTTCCCTTGCTCGATCTCCT |  |
| NM_174501.2 | *ALOX15* | F.1495 | AGGCCTGGTGTCGAGATATCA | 105 |
|  |  | R.1599 | TGGTCACAAAGTGGCAAAGC |  |
| XM_592026.7 | *CASP1* | F.193 | AGTGCTGAACCAGGAGGAGA | 188 |
|  |  | R.380 | CAGACTGTGAACCTGAAGTGAG |  |
| NM_174091.2 | *IL18* | F.366 | GACTGTTCAGATAATGCACCCC | 126 |
|  |  | R.491 | GTTCTCACAGGAGAGAGTAGAC |  |
| NM_174093.1 | *IL1B* | F.30 | ATTCTCTCCAGCCAACCTTCATT | 100 |
|  |  | R.129 | TTCTCGTCACTGTAGTAAGCCATCA |  |
| NM_001206735.1 | *IL1R* | F.1133 | AGGAGTACAGTCCTCGCACG | 144 |
|  |  | R.1276 | CTTCTGGAAATCGGGGACTGG |  |
| NM_173921.2 | *IL4* | F.266 | CACAGAACAGGTCTTGCTTGC | 122 |
|  |  | R.387 | AAACCTTCTGCAGGGTTGGA |  |
| NM_173923.2 | *IL6* | F.190 | CCAGAGAAAACCGAAGCTCTCAT | 100 |
|  |  | R.289 | CCTTGCTGCTTTCACACTCATC |  |
| NM_174088.1 | *IL10* | F.171 | GAAGGACCAACTGCACAGCTT | 98 |
|  |  | R.268 | AAAACTGGATCATTTCCGACAAG |  |
| NM_001110785.3 | *IL6R* | F.570 | GCTCTTTCTACGTATTGTCCCTGTGT | 100 |
|  |  | R.669 | GGGTCGGGCTGTAGGAGTTT |  |
| NM_001040555.1 | *IRAK1* | F.950 | CCTCAGCGACTGGACATCCT | 103 |
|  |  | R.1052 | GGACGTTGGAACTCTTGACATCT |  |
| NM_001075998.1 | *IRAK4* | F.54 | CGCCCGGGCAGGAATAAAAT | 133 |
|  |  | R.186 | GCGACTGCTAACTTCTTCCATC |  |
| NM_001102219.1 | *NLRP3* | F.69 | CTTTCTGGACTCTGACCGGG | 149 |
|  |  | R.228 | ATTGAGGTGCAGCCCTTCTG |  |
| NM_173966.3 | *TNF* | F.174 | CCAGAGGGAAGAGCAGTCCC | 114 |
|  |  | R.287 | TCGGCTACAACGTGGGCTAC |  |
| NM_174674.2 | *TNFRSF1A* | F.936 | CTGGTGATTGTCTTCGGGCT | 104 |
|  |  | R.1039 | TGCCCGCAAATGATGGAGTA |  |
| ^1^ Primer direction (F – forward; R – reverse) and hybridization position on the sequence. are underlined.  ^2^ Amplicon size in base pair (bp). | | | | |
