# Supplementary material for: Gene network expression of whole blood leukocytes in dairy cows with different milk yield at dry-off
Source: PLoS One. 2021 Dec 9;16(12):e0260745. doi: 10.1371/journal.pone.0260745 (PMC8659302; doi:10.1371/journal.pone.0260745)
Supplement: S5 Table — Sequencing results obtained from PCR product of Bos taurus specific primers for genes under investigation. (DOCX) [file pone.0260745.s006.docx]

| **S5 Table. Genes sequences.** Sequencing results obtained from PCR product of *Bos taurus* specific primers for genes under investigation. | |
| --- | --- |
| **Gene** | **Sequence** |
| *CCR2* | GTGGGCAACCTGCTGGTTGTCCTTATCCTAATCAACTGCAAAAAGCTGAAGAGCATGACTGACATCTACCTGCTTAACTTGGCCATCTCTGACCGCTGTTCCTCCTCACCATGCCGTTCTGGG |
| *CD44* | CAACTGTGCACCCGTCCCCAGACCAAGACGGCCCCTGGGTCTCCAGCGAGCCAGAGAATACCTCGGATACCAGAGACTACGGCA |
| *CX3CR1* | ACTTCGAGTATTATGACCTTGCAGAAGTCTGTGATATGGGGGACATCGTGGCCTTGGGAACTGTCTTCGTGGTCATACTCTACTCCCTCGTCTTTGCCTTTGGCCTGG |
| *ITGAL* | CAGGGGGCCGTGTACATCTTCAATGGGCAACAAGGGGGGCTGAGCCCCCGGCCCAGTCAGCGGATAGAAGGGACC |
| *ITGB2* | ATCGAATCGCAGGTGGACAGCCCAGAGGGACTGCGACGGCGTCCAGATCAACGTCCCGATCACCTTCAAGAGGG |
| *MMP9* | CGAGTTTGGCCACGCGCTGGGCTTAGATCACACCTCCGTGCCAGAGGCGCTCATGTACCCCATGTACAGATTCACAGAGGAGCACCCCA |
| *SELL* | ACGGGCCATGGACAATGTGTGGAAGTCATCAATAATTACACCTGCGTGCCCGTGATTTGGGGGGACCTCCGGAA |
| *SELPLG* | CTGGCAGCAGCCACCAGCTCGGGGGAGACCAGCACGAATGAAACTGTGAAGGCCCCAGTA |
| *TLN1* | ACCCCGTCCGCAATTCCTTCTCTACGTGCAGGCACGAGATGCCTAGGAAATGGCTCCCATCCTGTCTCCTCTGCTAAAGGCC |
| *TLN2* | ACCCCGTCCGCAATTCCTTCTCTACGTGCAGGCACGAGATGCCTAGGAAATGGCTCCCATCCTGTCTCCTCTGCTAAAGGCC |
| *CD14* | ACTGCAGCAGTGGCTCAAGCCTGGGCTCAGGGTGCTGAACATTGCCCAAGCACACTCA |
| *CD16* |  |
| *LGALS8* | AGCTTGGAACGTTGATTGTACTACGTGGGCATGTTCCTAGTGACTCGGACAGGTTCCAGA |
| *MYD88* | GACGCGGAGCATCGTAGAGGCCTTACGGTGGACTCTATAGACAGGCAGCATAACTCGGATAAATGGACATGGGCAACAC |
| *LCN2* |  |
| *MPO* | AGCCATGGGCTTAACATCACTTACCGGGATTACCTCGATATGGTGCTGGGGCGGGAGGCCCTAGAGGAAGTACCTGCGCGTA |
| *SOD1* | GTCCAAAAACCGGTGGGCCAAAAGATGAAGAGAGGCATGTTGGAGACCTGGGCAATGTGACAGCT |
| *SOD2* | GCATGTTTGGCCGATTATCTGAGGCCATTTTGGAATGTGATCAACTGGGAGAATGTAACTGCAATAC |
| *S100A8* | GTGTGCCATTAACTCCCTGATTGACGTCTACCACAAGTACTCCCTGAAAAAAGGGAATTACCACGCC |
| *IDO1* | ACCACATTGATGAAGACGTAGGCTTTGCTCTTCCATATCCACTGGAGGACCTACCTCATCC |
| *TLR2* | TTAAAAGAGTCACAATAGAAAGCTTAAGGTTTTTCTGGTTCCTTGTTTCACAACATTTAAATTCGGCGTTAGAATATTTGGATCGTCATAAAAACTTA |
| *YWHAZ* |  |
| *ACTB* | CACTTCTACACGAGCTGCGTGTGGCCCCCTGAGGAGCACCCCGTGCTGCTGACCGAGGCCCCCTCTGAACCCCAAGGCCAACCGTGAGAAGATGACCCAGATCATGTTCAGAGACA |
| *SDHA* |  |
| *ALOX5* |  |
| *ALOX15* | CTGTGATGATCCAGCGCTGTCTGAAGCTGCCAGACAATCTGCCGGTGACCACAGAGATGGTGGAGTGCAGCCTGGAGCGGCAGCTCACCCTGGAGCAGGAGATCGAGCAAGGAGAA |
| *CASP1* |  |
| **S5 Table (cont.)** | |
| **Gene** | **Sequence** |
| *IL18* | TGTATAAGGACAGCCTCACTAGAGGTCTGGCCGTAACCATCTCTGTGCAGTGTAAGAAAATGTCTACTCTCTCCTGTGAGAA |
| *IL1B* | ACAGCCATGGCACCGTACCTGAACCCATCAACGAAATGATCGGCTTACGTCACAGTGGACAGAGCACAATAGCACCCCC |
| *IL1R* |  |
| *IL4* |  |
| *IL10* | GCACGAGTACTCTCGTCACTAGGAGTACCTTTAAGGGTTACCTGGGTGTGCCAAGGCCTTGTCCGGAAATGATCCAGTTTACGA |
| *IL8* | AAAATCCTTTTTCCATTGCTTCTAAGAATTCCTCAGTAAAGATGCCAATGAAACTTCAAAACAAATCTACTTCAGTGCCTCAGTTCTGTGTGGGTCTGG |
| *IL6* | CAGAGAAGCTAGTAAGCTCTCATTAAGCGCATGGTCGACAACGTATTGCAATGAGAAAGGAGGCTAGTGAGAAGAATGATGA |
| *IL6R* | AGGTCGGGAACAAGTCCAGCAACCCCCTAGGATTTGACGGCTACAAACTCCTACAGCCCGACCCAA |
| *IRAK1* | GCTAGCGGGCATCTAGTTCTTACATCAAGGAATAGCCCCAGCCCTCATCCATGGAGAATGTCAAGAAGTTCCAACGCTCCAAAAAGGG |
| *NLRP3* | CTGGCTCGTTACCTGGTAGGACCTGGAAGACATAGACTTTAAGAAATTCAAGATGCATTTAGAAGACTATCCCAGTCAGAAGGGCTGCACCTCAAT |
| *TNF* | TCACTCTCCGGGGCAGCTCCGGTGGTGGGACTCGTATGCCAATGCCCTCATGGAA |
| *TNFRSF1A* | TCTGTCGTCTTAGCATGTCGCTACCAGCGGTGGAAGCCCAAGCTCTACTCCATCATTTGC  GGGCAA |
