# Supplementary material for: Gene network expression of whole blood leukocytes in dairy cows with different milk yield at dry-off
Source: PLoS One. 2021 Dec 9;16(12):e0260745. doi: 10.1371/journal.pone.0260745 (PMC8659302; doi:10.1371/journal.pone.0260745)
Supplement: S6 Table — Least square means of white blood cell populations percentage from -7 days from dry-off (DFD) to 34 DFD. (DOCX) [file pone.0260745.s007.docx]

| **S6 Table. White blood cell percentages.** Least square means of white blood cell populations percentage from -7 days from dry-off (DFD) to 34 DFD | | | | | | | |
| --- | --- | --- | --- | --- | --- | --- | --- |
|  | DFD | | |  | *P*-value^2^ | | |
| Item, unit | -7 | 7 | 34 | SEM^1^ | My | Day | My*Day |
| Neutrophils, % | 52.79 | 49.56 | 52.27 | 2.61 | 0.71 | 0.21 | 0.51 |
| Lymphocytes, % | 36.07 | 37.09 | 35.76 | 3.12 | 0.89 | 0.72 | 0.21 |
| Monocytes, % | 8.43 | 6.79* | 8.39 | 0.55 | 0.71 | <0.01 | 0.23 |
| Eosinophils, % | 1.68 | 5.52* | 2.71 | 1.22 | 0.52 | 0.03 | 0.86 |
| Basophils, % | 1.03 | 1.03 | 0.84 | 0.11 | 0.53 | 0.37 | 0.60 |
| * Significant difference (*P* < 0.05) with the reference (-7 DFD) | | | | | | | |
| ^1^ Greatest standard error of the mean | | | | | | | |
| ^2^ *P*-values of main effects: milk yield at dry-off (My), day, and interaction of milk yield × day (My*Day) | | | | | | | |
